# Gα12 and Gα13 proteins are required for transforming growth factor-β-induced myofibroblast differentiation

**DOI:** 10.1042/BCJ20240317

**Published:** 2024-12-13

**Authors:** Eleanor B. Reed, Albert Sitikov, Kun Woo D. Shin, Robert B. Hamanaka, Rengül Cetin-Atalay, Gökhan M. Mutlu, Alexander A. Mongin, Nickolai O. Dulin

**Affiliations:** 1 Department of Medicine, Section of Pulmonary and Critical Care Medicine, The University of Chicago, Chicago, IL, U.S.A.; 2 Department of Neuroscience and Experimental Therapeutics, Albany Medical College, Albany, NY, U.S.A.

**Keywords:** G proteins, myofibroblast differentiation, transforming growth factors

## Abstract

Myofibroblast differentiation, characterized by accumulation of cytoskeletal and extracellular matrix proteins by fibroblasts, is a key process in wound healing and pathogenesis of tissue fibrosis. Transforming growth factor-β (TGF-β) is the most powerful known driver of myofibroblast differentiation. TGF-β signals through transmembrane receptor serine/threonine kinases that phosphorylate Smad transcription factors (Smad2/3) leading to activation of transcription of target genes. Heterotrimeric G proteins mediate distinct signaling from seven-transmembrane G protein coupled receptors, which are not known to be linked to Smad activation. We tested whether G protein signaling plays any role in TGF-β-induced myofibroblast differentiation, using primary cultured human lung fibroblasts. Activation of Gαs by cholera toxin blocked TGF-β-induced myofibroblast differentiation without affecting Smad2/3 phosphorylation. Neither inhibition of Gαi by pertussis toxin nor siRNA-mediated combined knockdown of Gαq and Gα11 had a significant effect on TGF-β-induced myofibroblast differentiation. In contrast, combined knockdown of Gα12 and Gα13 significantly inhibited TGF-β-stimulated expression of myofibroblast marker proteins (collagen-1, fibronectin, smooth-muscle α-actin), with siGα12 being significantly more potent than siGα13. Mechanistically, combined knockdown of Gα12 and Gα13 resulted in substantially reduced phosphorylation of Smad2 and Smad3 in response to TGF-β, which was accompanied by a significant decrease in the expression of TGF-β receptors (TGFBR1, TGFBR2) and of Smad3. Thus, our study uncovers a novel role of Gα12/13 proteins in the control of TGF-β signaling and myofibroblast differentiation.

## Introduction

Transforming growth factor-β (TGF-β) is a pleotropic cytokine with multiple cell-specific functions. TGF-β was originally called ‘transforming’ because it enhanced anchorage-independent growth of normal rat kidney (NRK) cells on soft agar (a commonly used assay for cell transformation) in response to TGF-α or epidermal growth factor [1,2]. Subsequently it was found that TGF-β inhibited anchorage-dependent growth of NRK cells and of multiple human tumor cell lines; and it has been recognized as an inhibitor of cell cycle progression and cell proliferation [3]. Through numerous studies, it is now established that TGF-β controls fundamental cellular processes such as cell proliferation, survival, hypertrophy, senescence, epithelial-to-mesenchymal transition, cell differentiation; and it is implicated in a variety physiological and pathological processes [4]. This study focuses on signaling mechanisms that mediate one of the functions of TGF-β — differentiation of fibroblasts to myofibroblasts.

Myofibroblasts are phenotypically modified fibroblasts, originally characterized by the presence of a well-developed contractile apparatus and the formation of robust actin stress fibers containing the smooth muscle α-actin (SMA) isoform normally expressed in smooth muscle cells [5,6]. Myofibroblasts produce extracellular matrix proteins such as fibronectin (FN), multiple isoforms of collagen and other proteins implicated in matrix remodeling [7–9]. Over decades of research, myofibroblasts have been recognized as the key cells in wound healing and pathogenesis of tissue fibrosis [10,11].

TGF-β is the most powerful known driver of myofibroblast differentiation [12]. TGF-β signals through transmembrane-receptor serine/threonine kinases that phosphorylate Smad transcription factors (Smad2/3), leading to their heteromerization with a common mediator Smad4, nuclear translocation of the Smad2/3/4 complex and activation of transcription of target genes [13,14]. G protein coupled receptors (GPCRs), the largest receptor family regulating various functions of all mammalian cells, transduce extracellular signals through heterotrimeric G proteins, with Gα and Gβγ subunits controlling the activity of specific target proteins [15]. Four functionally distinct types of Gα subunits have been identified: Gαs, Gαi, Gαq/11, and Gα12/13 [16]. Gαs activates adenylyl cyclase to produce cAMP, whereas Gαi inhibits this enzyme [17]. Gαq/11 activate phospholipase Cβ [18,19] generating two second messengers — inositol trisphosphate and diacylglycerol — that stimulate calcium release from endoplasmic reticulum and activate protein kinase C, respectively [20]. In addition, Gβγ proteins can also activate PLCβ isoforms [21]. Gα12/13 stimulate the Rho family of small GTPases through direct recruitment of specific guanine exchange factors (GEFs) for RhoA, a small GTPase [22–25].

Little is known about the cross-talk between TGF-β and G protein signaling in the context of myofibroblast differentiation. Agonists coupled to Gαs (prostaglandin E2, prostacyclin, adrenomedullin) have been shown by us and others to inhibit TGF-β-induced myofibroblast differentiation through a protein kinase A (PKA)-dependent mechanism [26–29]; however, the role of Gαs has not been evaluated in these studies. Better understanding of the role of Gαs is important, given that PKA can be stimulated through other mechanisms, including a Gβγ-mediated one [30]. GPCR agonists acting through Gαi, Gαq/11, and Gα12/13 (i.e. lysophosphatidic acid (LPA), sphyngosine-1-phosphate) have been reported to promote myofibroblast differentiation [31,32]; however, the role of specific G proteins was not identified. A link between TGF-β and G protein signaling has been reported, wherein GPCR ligands (angiotensin II, thrombin, etc.) promote TGF-β synthesis [33,34] or release of active TGF-β from the pericellular matrix [35]. However, a direct role of G proteins in TGF-β signaling in the context of myofibroblast differentiation has not been investigated. In this study, we sought to determine that role of G proteins, using primary cultured human lung fibroblasts (HLFs), and utilizing a knockdown approach of individual Gα subunits, activation of Gαs by cholera toxin (CTX) and inhibition of Gαi-Gβγ pathway by pertussis toxin (PTX).

## Results

### Activation of Gαs by CTX blocks TGF-β-induced myofibroblast differentiation without affecting Smad2/3 phosphorylation

**Figure 1.**
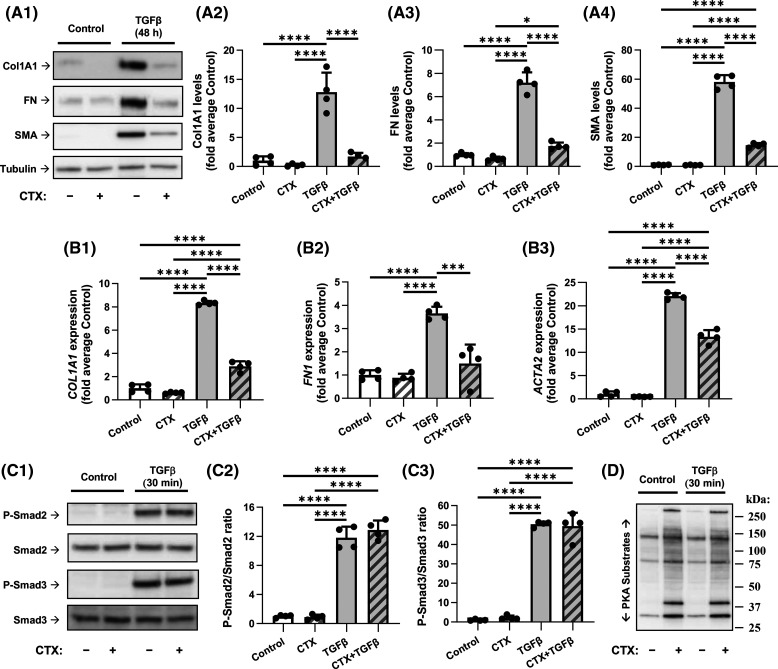
Activation of Gαs by cholera toxin blocks TGF-β-induced myofibroblast differentiation without affecting Smad2/3 phosphorylation. (**A**) Representative images and quantification of western blot analyses of myofibroblast markers in human lung fibroblasts (HLF). HLF were serum-starved for 48 h and then treated with vehicle or 1 µg/ml cholera toxin (CTX), immediately followed by treatment with vehicle or 1 ng/ml TGF-β for additional 48 h, as indicated. HFL lysates were analyzed by western blotting using antibodies recognizing collagen 1A1 (Col1A1, A2), fibronectin (FN, A3) and smooth muscle α-actin (SMA, A4). The relative immunoreactivity values were normalized to the average signal of control samples. (**B**) RT-qPCR analysis of myofibroblast markers in HLF treated with vehicle, 1 µg/ml cholera toxin (CTX), and/or 1 ng/ml TGF-β (as in **A**) for 24 h. mRNA levels of *COL1A1* (B1), *FN1* (B2), and *ACTA2* (B3) were normalized within-the-sample to the levels of housekeeping ribosomal RPL13 mRNA and compared with an average expression in control samples. (**C**) Representative images and quantification of western blot analyses of Smad2/3 phosphorylation in HLF pretreated with vehicle or 1 µg/ml CTX for 2 h followed by 30-min exposure to vehicle or 1 ng/ml TGF-β. Cell lysates were analyzed by western blotting with antibodies recognizing Smad2/3 and their phosphorylated forms as indicated. pSmad/Smad ratios were quantified for Smad2 (C2) and Smad3 (C3). (**D**) Western blot analysis of PKA-dependent phosphorylation in HFL treated as in **C***.* and then probed with antibody recognizing phosphorylated PKA substrates (representative of three experiments). Quantitation data in **A–C** are the mean values ± SD of four independent treatments per group. *P < 0.05, ***P < 0.001, ****P < 0.0001, one-way ANOVA with Tukey correction for multiple comparisons.

Previous studies by us and others demonstrated regulation of TGF-β-induced myofibroblast differentiation by agonists acting in part through Gαs-coupled GPCRs [26–29]; however, a direct role of Gαs has not been carefully investigated. CTX, which ADP-ribosylates and inhibits the GTPase activity of Gαs, is recognized as a powerful and highly specific activator of Gαs [36]. Therefore, we used CTX as a tool for investigating the effect of Gαs activation on TGF-β-induced myofibroblast differentiation. As shown in Figure 1A, CTX significantly reduced TGF-β — induced accumulation of the myofibroblast marker proteins collagen 1A1 (Col1A1), FN and SMA, when applied immediately following TGF-β treatment for 48 h (a time point at which near maximum accumulation of these proteins occurs in response to TGF-β). CTX also significantly reduced mRNA levels of *Col1A1*, *FN1* and *ACTA2* (for SMA protein) after 24 h of exposure to TGF-β (a time point previously identified for maximum increase in mRNA levels for these genes in response to TGF-β) (Figure 1B). Pretreatment of HLF with CTX for 2 h (2 h is sufficient for ADP-ribosylation to occur in cells) did not affect Smad2/3 phosphorylation induced by acute (30 min) TGF-β treatment (Figure 1C). Activation of Gαs 2 h post treatment with CTX was confirmed by western blotting with ‘protein kinase A (PKA) substrate’ antibodies (Figure 1D) that recognize proteins phosphorylated by PKA (a downstream effector of Gαs) and whose specificity was previously demonstrated through the expression of a specific PKA inhibitor protein, PKI [37]. Two-hour treatment of HLF with CTX resulted in an obvious change in morphology of the cells, that is characteristic of reduced assembly of actin stress fibers and is similar to a known effect of PKA activation in fibroblasts [38]. This morphological change induced by CTX was partially prevented by knockdown of Gαs (Supplementary Figure S1). Supplementary Figure S2 confirms efficient knockdown of both Gαs-long and Gαs-short isoforms by western blotting; and, importantly, it demonstrates that Gαs knockdown partially prevents inhibition of TGF-β-induced myofibroblast differentiation by CTX. Interestingly, long-term (48-h) CTX treatment also resulted in a significant down-regulation of Gαs proteins, the mechanism of which will be further investigated. Finally, TGF-β-induced myofibroblast differentiation was inhibited by an activator of adenylyl cyclase, forskolin (Supplementary Figure S3). Together, these data demonstrate that Gαs activation results in inhibition of TGF-β-induced myofibroblast differentiation without affecting proximal TGF-β signaling.

### Inhibition of Gαi by PTX does not affect TGF-β-induced myofibroblast differentiation

**Figure 2.**
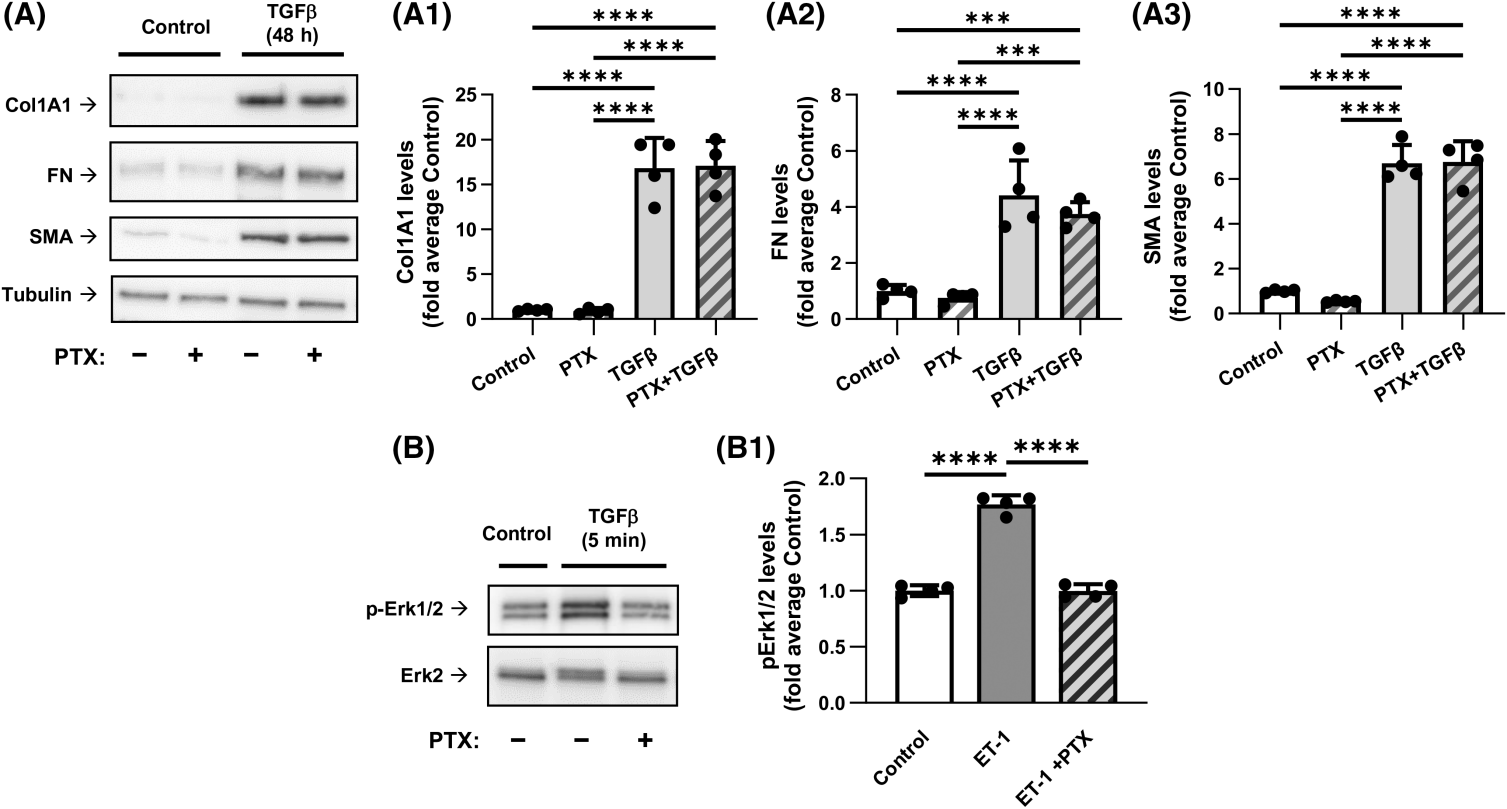
Inhibition of Gαi by pertussis toxin does not affect the TGF-β-induced myofibroblast differentiation. (**A**) Representative images and quantification of western blot analyses of HLF pretreated overnight with 100 ng/ml pertussis toxin (PTX), followed by treatment with either vehicle or 1 ng/ml TGF-β for 48 h. HLF lysates were analyzed using antibodies recognizing Col1A1 (**A1**), FN (**A2**), and SMA (**A3**). The relative luminescence values were normalized to the average values of controls. Data are the mean values ± SD from four independent cultures per treatment. ***P < 0.001; ****P < 0.001, one-way ANOVA with Tukey correction for multiple comparisons. (**B**) Representative images of western blot analyses of the PTX pretreated HFL with or without subsequent 5-min treatment with 100 nM endothelin-1 (ET1). Cell lysates were probed with antibodies recognizing p-Erk1/2 or total Erk2. (**B1**) Quantification of experiments presented in **B**. ****P < 0.001, one-way ANOVA with Tukey correction for multiple comparisons.

We then focused on the role of Gαi, using PTX, which ADP-ribosylates and blocks the activity of Gαi through inhibition of GDP to GTP exchange by Gαi [39,40]. Pretreatment of HLFs with PTX had no significant effect on TGF-β-induced expression of Col1A1, FN and SMA (Figure 2A). We have previously established that Gαi-coupled Gβγ mediates phosphorylation of extracellular signal regulated kinases ERK1/2 by endothelin-1 (ET-1) in vascular smooth muscle cells [30]. Therefore, we confirmed that PTX was effective in the inhibition of Gαi in HLFs by demonstrating that PTX pretreatment abolished ET1-induced phosphorylation of ERK1/2 (Figure 2B). Together, these data suggest that Gαi may not be involved in TGF-β-induced myofibroblast differentiation.

### Knockdown of Gαq/11 does not affect TGF-β-induced myofibroblast differentiation

**Figure 3.**
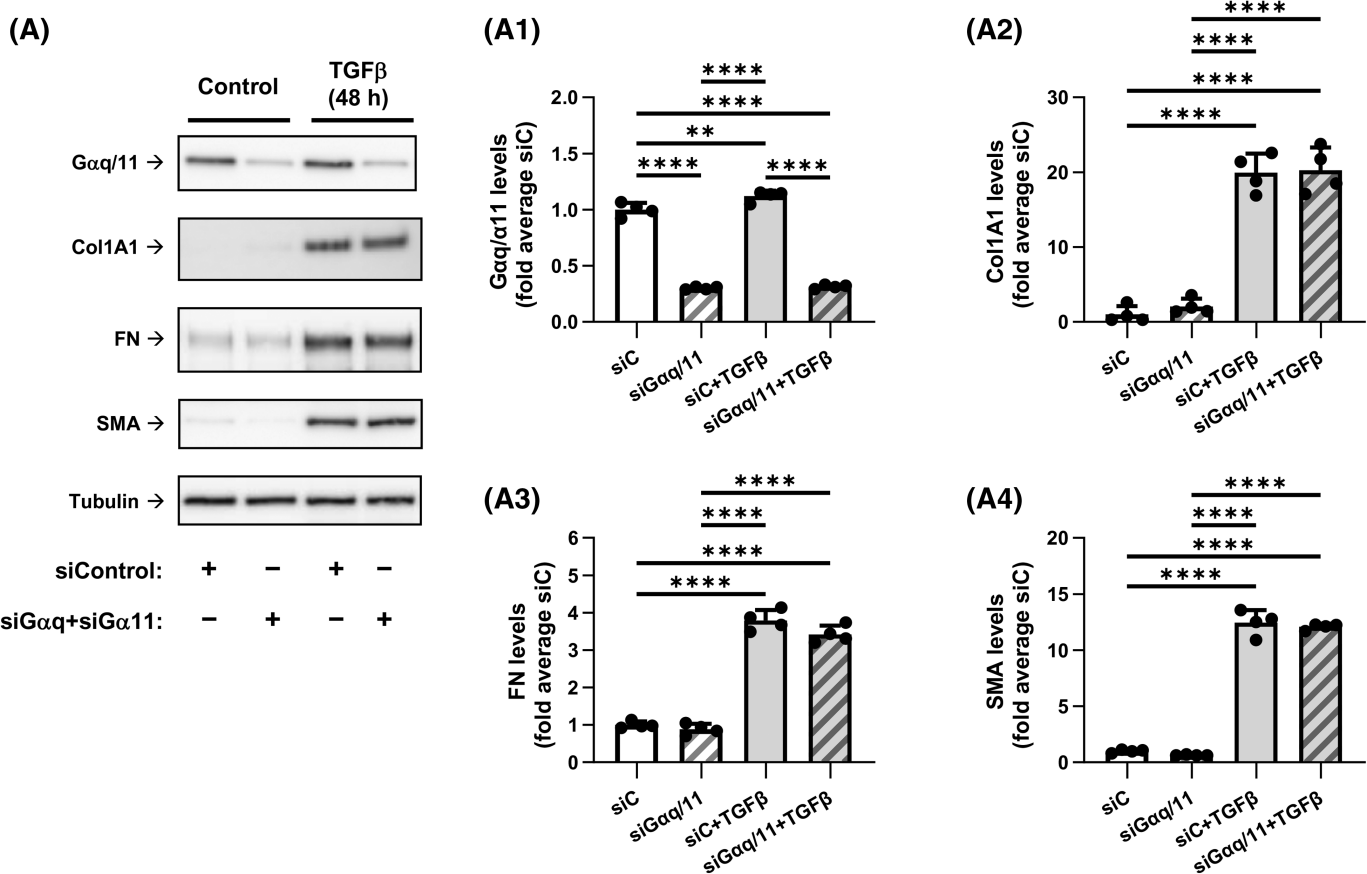
Knockdown of Gαq and Gα11 does not affect the TGF-β-induced myofibroblast differentiation. (**A**) Representative images and quantification of western blot analyses of HLF transfected overnight with either control siRNA (siC) or with siRNAs targeting Gαq and Gα11. HFL were next serum starved for 48 h, followed by the treatment with either vehicle or 1 ng/ml TGF-β for 48 h. Cell lysates were analyzed using antibodies recognizing Gαq/Gα11 (**A1**), Col1A1 (**A2**), FN (**A3**), and SMA (**A4**). The relative luminescence values were normalized to the average siC-treated control samples. Data are the mean values ± SD from four independent cultures per treatment. **P < 0.01; ****P < 0.001, one-way ANOVA with Tukey correction for multiple comparisons.

To assess the role of Gαq/11, we used the siRNA approach. Combined knockdown of Gαq and Gα11 with corresponding siRNAs resulted in a 70% decrease in the expression of each protein in the presence or absence of TGF-β, as assessed by western blotting with antibodies that recognize both Gαq and Gα11 (Figure 3). Under the same treatment conditions, Gαq/11 knockdown had no significant effect on TGF-β-induced myofibroblast differentiation, suggesting a possible lack of the role of Gαq/11 in this process.

### Knockdown of Gα12 and Gα13 attenuates TGF-β-induced myofibroblast differentiation in a synergistic fashion

**Figure 4.**
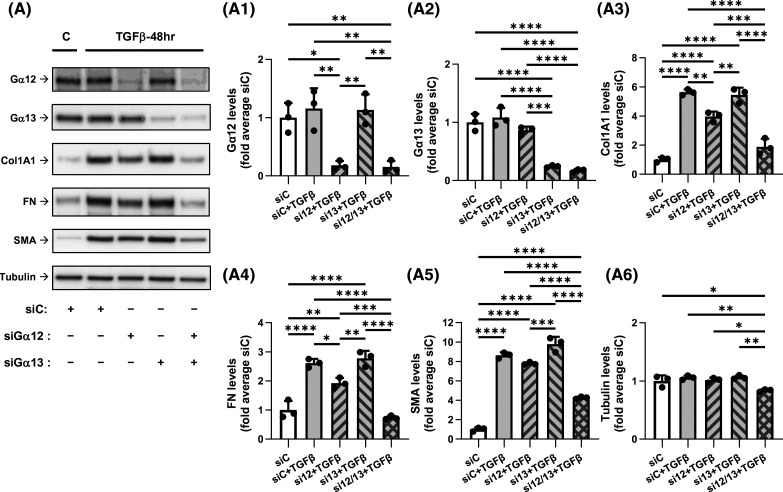
Knockdown of Gα12 and Gα13 attenuates the TGF-β-induced myofibroblast differentiation in a synergistic fashion. (**A**) Representative images and quantification of western blot analyses of HLF transfected overnight with control siRNA (siC, 10 nM), or combination of siC (5 nM) plus siGα12 (5 nM), or siC (5 nM) plus siGα13 (5 nM), or siGα12 (5 nM) plus siGα13 (5 nM). Cells were next serum starved for 48 h, and further treated with either vehicle or 1 ng/ml TGF-β for additional 48 h. HFL lysates were analyzed by western blotting using antibodies recognizing Gα12 (**A1**), Gα13 (**A2**), Col1A1 (**A3**), FN (**A4**), SMA (**A5**), or tubulin **(A6**). Data are the mean values ± SD from three independent cultures per treatment. *P < 0.05, **P < 0.01; ***P < 0.001; ****P < 0.001, one-way ANOVA with Tukey correction for multiple comparisons.

We then examined the roles of Gα12 and Gα13 in TGF-β-induced myofibroblast differentiation also using an siRNA approach. As shown in Figure 4, knockdown of Gα12 and of Gα13 achieved up to 85% and 80% reductions in corresponding protein expression levels in HLFs. Knockdown of Gα12 resulted in a moderate inhibition of TGF-β-induced expression of Col1A1, FN and SMA by 30%, 25%, and 10%, respectively. Knockdown of Gα13 alone had no significant effect on the expression of these proteins. However, combined knockdown of Gα12 and Gα13 significantly potentiated the effect of individual Gα12 knockdown, decreasing TGF-β-induced protein expression of Col1A1, FN and SMA by 80%, 100%, and 60%, respectively (Figure 4). TGF-β treatment resulted in accumulation of SMA-positive stress fibers which was attenuated by Gα12/Gα13 knockdown, as determined by immunofluorescent microscopy of SMA (Supplementary Figure S4). Gα12/Gα13 knockdown also reduced the induction of mRNA levels of *Col1A1*, *FN1* and *ACTA2* in response to TGF-β (Supplementary Figure S5), suggesting control of their expression by Gα12/Gα13 at a transcriptional level. Inhibition of TGF-β-induced myofibroblast differentiation by combined knockdown of Gα12 and Gα13 was further confirmed using an alternative set of siRNAs against these genes (Supplementary Figure S6). Together, these data indicate that both Gα12 and Gα13 proteins are required for full TGF-β-induced myofibroblast differentiation, with Gα12 being potentially of higher importance.

### Combined knockdown of Gα12 and Gα13 inhibits TGF-β-induced Smad2/3 phosphorylation

**Figure 5.**
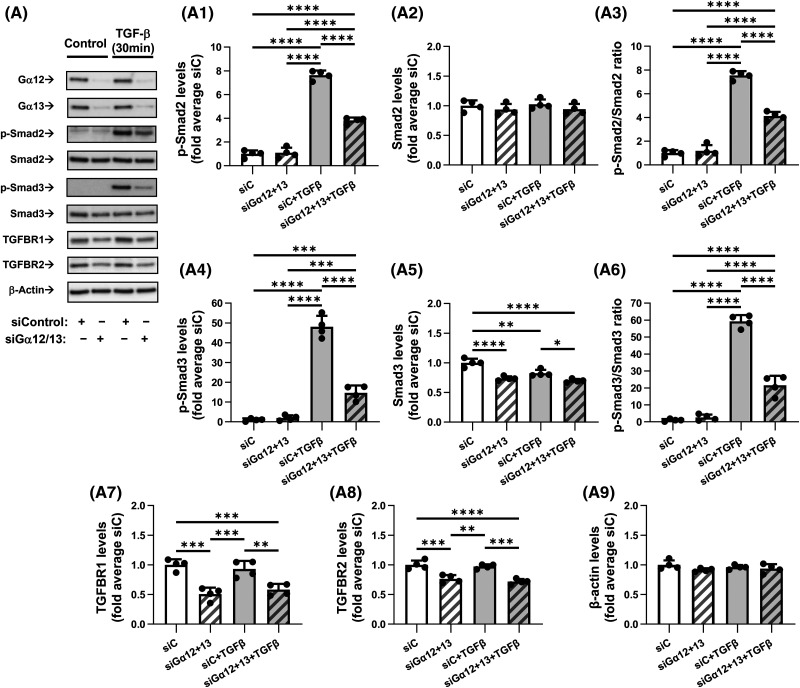
Combined knockdown of Gα12 and Gα13 inhibits TGF-β-induced Smad2/3 phosphorylation and reduces TGFβ receptor levels. (**A**) Representative images and quantification of western blot analyses of HLF transfected overnight with control siRNA (siC, 10 nM) or with a combination of siRNAs targeting Gα12 (5 nM) and Gα13 (5 nM). HLF were next serum starved for 48 h and additionally treated with either vehicle or 1 ng/ml TGF-β for 30 min. Protein lysates were analyzed by western blotting using antibodies against p-Smad2, Smad2, p-Smad3, Smad3, TGFBR1, TGFBR2, or β-actin. Quantifications show chemiluminescence levels of p-Smad2 (**A1**), Smad2 (**A2**), ratios of p-Smad2/Smad2 (**A3**), p-Smad3 (**A4**), Smad3 (**A5**), ratios of p-Smad3/Smad3 (**A6**), TGFBR1 (**A7**), TGFBR2 (**A8**) and β-actin (**A9**), normalized to actin levels and average of siC. Data are the mean values ± SD from four independent cultures per treatment. *P < 0.05; **P < 0.01; ***P < 0.001; ****P < 0.001, one-way ANOVA with Tukey correction for multiple comparisons.

To begin understanding the mechanism by which Gα12/13 control TGF-β-induced myofibroblast differentiation, we tested the effect of combined Gα12/13 knockdown on TGF-β-induced phosphorylation of Smad2 and Smad3 — an initial event in TGF-β receptor signaling. As shown in Figure 5, Gα12/13 knockdown significantly inhibited TGF-β-induced phosphorylation of both Smad2 and Smad3, as assessed by Western blotting with corresponding phospho-Smad antibodies. The total levels of Smad2 were not significantly affected, whereas Smad3 expression was significantly reduced by up to 25% under Gα12/13 knockdown conditions. Normalized data revealed a substantial and highly significant reduction of P-Smad2/Smad2 (45%) and P-Smad3/Smad3 (64%) ratios under siGα12/13 treatment. These data suggest that the decrease in Smad2/3 phosphorylation could not be explained solely by a reduction of Smad2/3 levels. Therefore, we next examined TGF-β receptor levels and observed highly significant reductions in the expressions of TGFBR1 (37%) and (to a lesser extent) TGFBR2 (26%) in HLFs treated with siGα12/13 as compared with HLFs treated with control siRNA (Figure 5). Together, these data suggest that Gα12/13 regulate TGF-β-induced Smad2/3 phosphorylation by controlling the expression of TGF-β receptors and Smad3.

## Discussion

The major finding of this study is the discovery that Gα12/13 proteins mediate TGF-β-induced myofibroblast differentiation, at least in part through control of proximal TGF-β signaling (Smad2/3 phosphorylation) (Figures 4 and 5). An important question is whether TGF-β can indirectly activate G protein signaling, specifically that of Gα12/13. We and others have previously demonstrated that TGF-β, through Smad-dependent gene transcription, recruits RhoA signaling, actin polymerization and activation of a transcription factor, serum response factor, for the induction of SMA expression in fibroblasts [28,41,42]. Given that the RhoA pathway is activated by Gα12/13 [22–25], it is reasonable to propose that Gα12/13 are activated at some point through TGF-β signaling. This notion is supported by our finding that Gα12/13 knockdown also abolished TGF-β-induced phosphorylation of myosin light chain (Supplementary Figure S7), which is controlled by Rho-mediated signaling [43] and has been commonly used as indirect assay for RhoA activation [44–46]. Another question is: which GPCRs may be activated during TGF-β signaling. Some candidate GPCRs are worthy of consideration based on current reports. It was shown that LPA, sphingosine-1-phospphate (S1P) and thrombin co-operate in human dermal fibroblasts with TGF-β to induce extracellular matrix synthesis, myofibroblast marker expression and cytokine secretion [47]. Sphingosine-1 phosphate (S1P) receptor signaling was shown to be important for TGF-β-induced myofibroblast differentiation in a number of studies [48–50]. Last but not least, TGF-β induces ET1 expression [51], although it may also down-regulate ET1 receptors [52]. More than 30 GPCRs have been reported to couple to Gα12/13 [53]; hence, identification of critical GPCRs mediating TGF-β signaling related to myofibroblast differentiation requires further investigation and is of potential therapeutic importance for treatment of tissue fibrosis.

We also observed that while combined knockdown of Gα12/13 abolished TGF-β-induced myofibroblast differentiation, knockdown of Gα12 alone had a significant (though partial) inhibitory effect whereas knockdown of Gα13 had not (Figure 4). It is noteworthy that while both Gα12 and Gα13 are linked to RhoA activation, they may recruit different Rho GEFs [22–25] and they may couple to different GPCRs [54], the molecular mechanisms of which have been under investigation [55]. Our studies have not revealed a role of Gαi and Gαq/11 in TGF-β-induced myofibroblast differentiation (Figures 2 and 3). This, however, does not negate the significance of Gαi and Gαq/11 for fibroblast biology, given the established importance of Gαi/Gβγ and Gαq/11 signaling in cell proliferation, migration and contraction — all critical for the function of fibroblasts in wound healing and pathogenesis of tissue fibrosis. Finally, inhibition of TGF-β-induced myofibroblast differentiation by CTX or forskolin (Figure 1, Supplementary Figures S1–S3) was intuitively expected given the reported inhibitory effects of cAMP-promoting agonists (i.e. prostaglandin E2, prostacyclin, adrenomedullin) [26–29]. However, to our knowledge, this is the first direct demonstration of regulation of TGF-β-induced myofibroblast differentiation by Gαs without affecting the proximal TGF-β signaling (Smad2/3 phosphorylation).

Our results suggest that a dependence of TGFBR1/2 and Smad3 expression on Gα12/13 (Figure 5) could be one mechanism by which Gα12/13 control TGF-β-induced myofibroblast differentiation, which will be evaluated in the future by forced overexpression of Smad3 and TGFBR1/2. The proteasome inhibitor MG-132 had no significant effect on TGF-β-induced expression of Col1A1 and SMA, and it even mildly decreased TGF-β-induced FN expression; but importantly, MG-132 did not reverse the inhibitory effect of Gα12/13 knockdown on the effects of TGF-β (Supplementary Figure S8). This suggests that proteasomal protein degradation is not the key mechanism for the regulation of TGF-β-induced myofibroblast differentiation by Gα12/13. Furthermore, Gα12/13 knockdown did not affect basal or TGF-β-induced mRNA levels of the mitochondrial metabolic genes *NDUFA4*, *SDHB* and *COX17* (Supplementary Figure S9) that we have previously investigated in relation to myofibroblast biology [56]. Thus, down-regulation of TGFBR1/2 and Smad3 expression resulting from Gα12/13 knockdown may not be the sole mechanism of regulation of myofibroblast differentiation by Gα12/13 proteins.

Other possibilities of regulation of Smad2/3 phosphorylation may exist, e.g. at the level of interaction of TGFBR1 with Smad2/3. For example, it was shown that the small GTPase RhoB (but not RhoA) interacts with Smad3, blocks the interaction of Smad3 with TGFBR1and prohibits its phosphorylation [57]. The mechanisms by which Gα12/13 control Smad2/3 signaling require further investigation.

In all, our study describes a novel cross-talk between TGF-β and G protein signaling in the context of myofibroblast differentiation and encourages new investigations on this cross-talk in other cellular functions of TGF-β.

## Materials and methods

### Primary culture of HLFs

HLFs were isolated from the lungs of patients with idiopathic pulmonary fibrosis shortly after their removal during lung transplantation at the University of Chicago, under IRB protocol #14514A as described previously [46]. Human lung tissue samples were placed in Dulbecco's Modified Eagle Medium (DMEM) with antibiotics. Lung tissue was minced to ∼1 mm^3^ pieces, washed, and plated on 10-cm plates in growth media containing DMEM supplemented with 10% FBS and antibiotics. The media was changed twice a week. After ∼2 weeks, the explanted and amplified fibroblasts were trypsinized, cleared from tissue pieces by sedimentation, and further amplified as passage 1. Unless indicated, cells were grown in growth media for 24 h, starved in DMEM containing 0.1% FBS for 48 h, and treated with desired drugs for various times as indicated in the figure legends. Primary cultures were used from passage 3 to 8.

### siRNA-mediated knockdown

HLFs were plated at a density of 0.4 × 10^5^ cells per well (24-well plates) and were grown for 24 h. Cells were then transfected with total of 10 nM desired siRNA using Lipofectamine RNAiMAX Reagent (ThermoFisher Scientific, Waltham, MA, U.S.A.) according to the standard protocol, and kept in growth media for additional 24 h, followed by serum starvation in DMEM with 0.1% FBS for 48 h, and then by treatment with TGF-β for desired times. siRNAs for *Gαs* (Hs_GNAS_4: AACCAAAGTGCAGGACATCAA), *Gαq* (HS_GNAQ_8: GACGACGAGAATATCAATTAT), *Gα11* (HS_GNA11_6: AGCGACAAGATCATCTACTCA), *Gα12* (HS_GNA12_2: CCGGATCGGCCAGCTGAATTA, Gα13 (HS_GNA13_1: CCCGACTGCTTACCAAATTAA, or control siRNA (1027281, sequence proprietary) were from Qiagen. Supplementary Figure S10 shows that a control siRNA (siC) had no significant effect on TGF-β-induced expression of Col1A1, although it inhibited this effect on FN and SMA by 25% and 20%, respectively. Therefore, all the siRNA experiments included siC as control.

### Western blotting

Western blotting was performed as described previously [46]. HLFs were lysed in a buffer containing 8M deionized urea, 1% SDS, 10% glycerol, 60 mM Tris–HCl, pH 6.8, 0.02% pyronin Y, and 5% β-mercaptoethanol. Lysates were sonicated for 5 s. Samples were then subjected to polyacrylamide gel electrophoresis and Western blotting with desired primary antibodies and corresponding horseradish peroxidase (HRP)-conjugated secondary antibodies and developed by chemiluminescence reaction. Digital chemiluminescent images below the saturation level were obtained with a LAS-4000 analyzer, and light intensity was quantified using Multi Gauge software (Fujifilm, Valhalla, NY, U.S.A.). Primary antibodies were validated by molecular mass of target proteins and by siRNA-mediated knockdown (Supplementary Figure S11).

### RNA isolation and quantitative PCR

RNA was isolated using the GenElute Total RNA Purification Kit (Sigma) and reverse transcribed using iScript Reverse Transcription Supermix (Bio-Rad). Quantitative mRNA expression was determined by real-time RT-PCR using ITaq Universal SYBR Green Supermix (Bio-Rad). The list of primers used for PCR is presented in Supplementary Figure S12.

### Immunofluorescence microscopy

Cells were grown on glass chamber slides, serum starved and treated with desired agonists for desired times. Cells were washed twice with ice-cold PBS, fixed in 4% paraformaldehyde in PBS for 15 min at room temperature, washed again with PBS, and permeabilized in 0.5% Triton-X100 in PBS for 5 min, followed by incubation with 1% bovine serum albumin and 5% goat serum in PBS for 1 h. Cells were then incubated with antibodies against SMA (1:300) in PBS/BSA overnight at 4°C, washed five times with PBS, followed by incubation with Alexa Flour™ 594 goat anti-mouse IgG (Invitrogen, A11032, 1–300) in PBS/BSA for 1 h at room temperature. The slides were additionally washed five times with PBS, and the coverslips were mounted using VECTASHIELD antifade mounting medium containing DAPI for staining of nuclei. Images were taken under Nikon Ti-2 fluorescent microscope.

### Materials

Recombinant TGF-β (T7039), CTX (227036) and PTX (516560) were from Millipore-Sigma. The following antibodies for Western blotting were from Millipore-Sigma: SMA (A5228, 10,000×), β-actin (A5441, 10,000×), α-tubulin (T6074, 10,000×). FN antibody (610077, 1000×) was from BD Transduction. Antibodies against human collagen-1A1 (sc-28657, 1000×), TGFBR2 (sc-400) were from Santa Cruz Biotechnology. Antibodies against Smad2 (L1603, 1000×), phospho-Smad2-Ser465/467 (138D4, 1000×), phospho-Smad3-Ser423/425, 1000×) were from Cell Signaling Technology. Gα12 antibody (GTX114147, 1000×) and Gα13 antibody (GTX32613, 1000×) were from GeneTex. Gαs antibody was from ABclonal (A5546, 1000×). Smad3 antibody (06-920, 1000×) was from Upstate Biotechnology. TGFBR1 antibody (AB235578, 1000×) was from Abcam. Secondary HRP-conjugated antibodies for western blotting (1:3000 dilution) were from Millipore-Sigma (40-139-32 — anti-rabbit IgG, 40-125-32 — anti-mouse IgG).

### Statistical analysis

In this study, a replicate (*n*) represents an independently plated and treated HLF culture. All individual data points are presented in figures along with mean values ± standard deviation (SD). Results were analyzed for normal distribution using a Shapiro-Wilk test. Normally distributed data were further statistically compared using one-way ANOVA with the Tukey honest significant difference post hoc correction for multiple comparisons. Values of *P* < 0.05 were considered statistically significant. All statistical analyses were performed in Prism v. 10.2.3 (GraphPad Software, Boston, MA, U.S.A.).

## Data Availability

Data obtained in this study are available upon request to the corresponding author.

## Abbreviations

Col1A1: collagen-1 isoform-A1
CTX: cholera toxin
DMEM: Dulbecco's Modified Eagle Medium
ET-1: endothelin-1
FN: fibronectin
GPCR: G protein coupled receptor
HLF: human lung fibroblasts
HRP: horseradish peroxidase
LPA: lysophosphatidic acid
NRK: normal rat kidney
PKA: protein kinase A
PTX: pertussis toxin
siRNA: short interfering RNA
SMA: smooth muscle α-actin
TGF-β: transforming growth factor-β.
