# Supplementary material for: Gα12 and Gα13 proteins are required for transforming growth factor-β-induced myofibroblast differentiation
Source: Biochem J. 2024 Dec 13;481(24):1937–48. doi: 10.1042/BCJ20240317 (PMC11668492; doi:10.1042/BCJ20240317)
Supplement: Supplementary Material 1 [file BCJ-481-1937-s1.pdf]

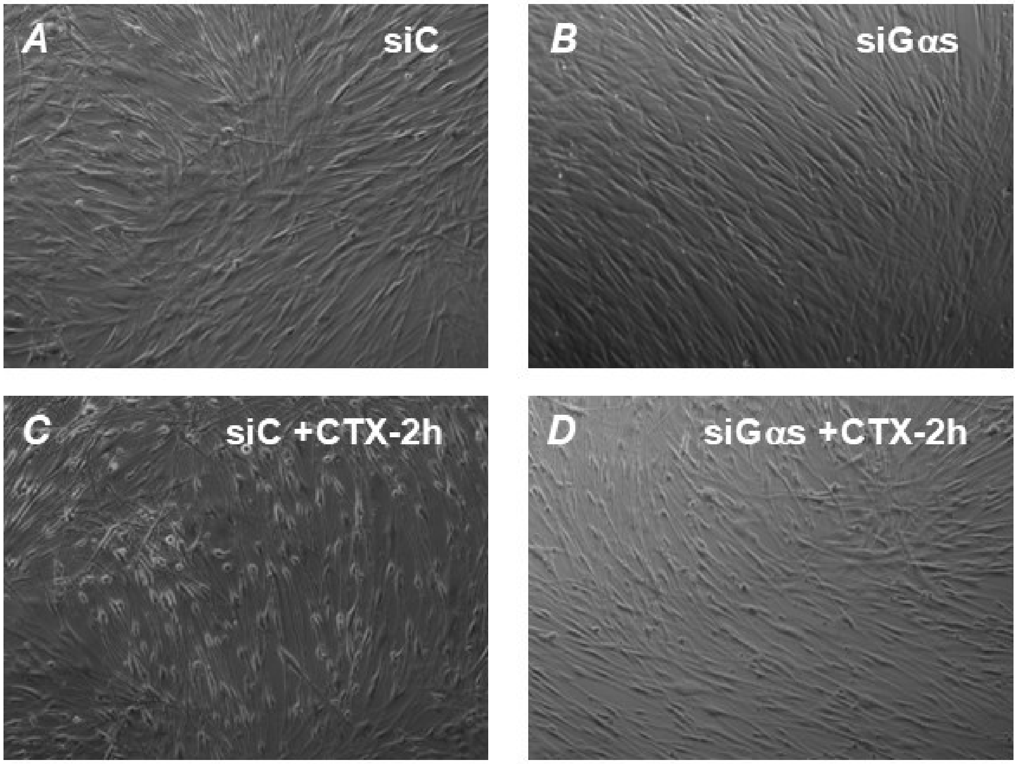

Cholera toxin (CTX) induces morphological change in HLF characteristic of disassembly of actin stress fibers, and this effect is partially rescued by Gαs knockdown

Representative phase contrast images of HLF transfected overnight with control siRNA (siC, 10 nM) or siRNA targeting GNAS (encoding Gαs, 10 nM). Cells were next serum-starved for 48 h and additionally treated for 2 h with either vehicle (***A***, ***B***) or 1 μg/ml CTX (***C***, ***D***). Images were taken using an Olympus IX-71 microscope with 10× lens magnification.

Supplemental Figure S2 ● E.B. Reed et al. New, ungrouped panels

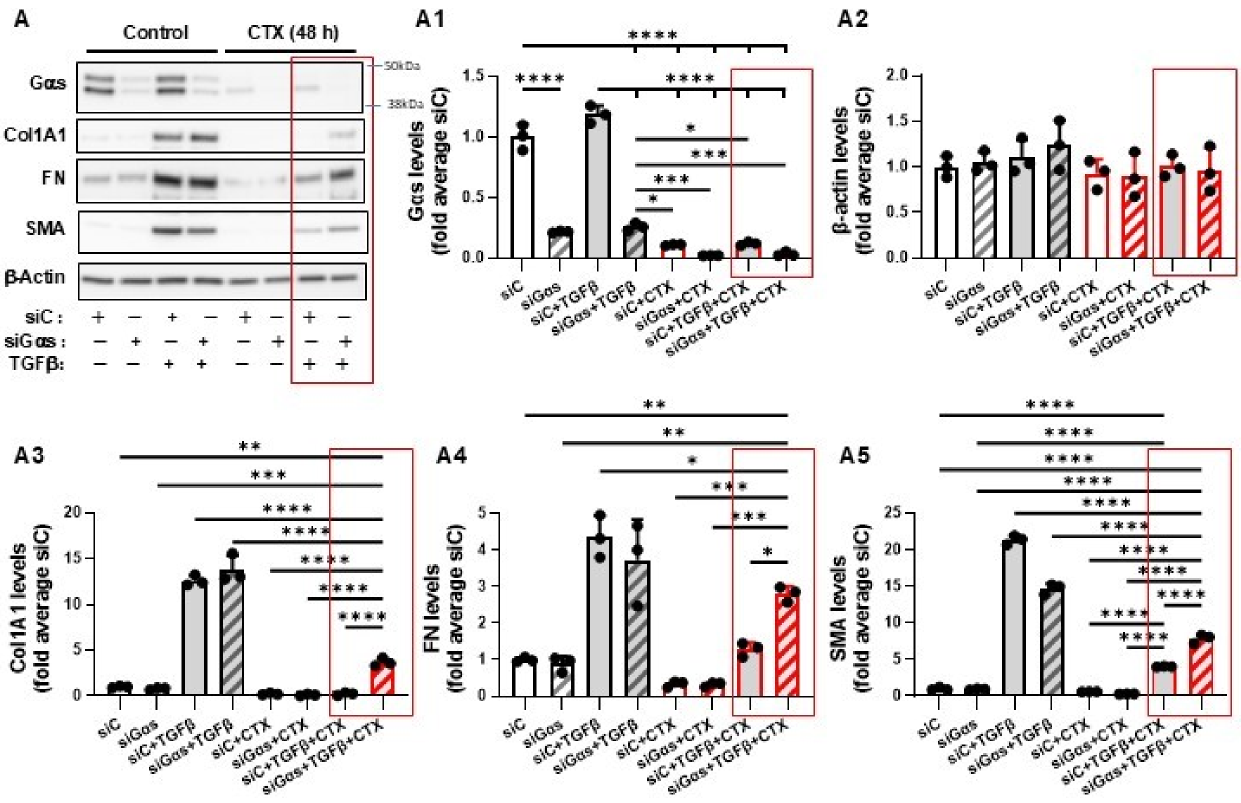

Knockdown of Gαs partially rescues inhibition of the TGF-β-induced myofibroblast differentiation by cholera toxin. HLF were transfected overnight with either control siRNA (siC) or siRNA targeting Gαs (siGαs). Cells were next serum starved for 48 hours, and then treated for additional 48 hours with vehicle, 1 μg/ml cholera toxin (CTX), and/or 1 ng/ml TGF-β as indicated. Cell lysates were analyzed with antibodies recognizing Gαs, Col1A1, FN, SMA and β-actin. Shown are representative western blot images (**A**) and quantifications of chemiluminescence signal for Gαs (**A1**), β-actin (**A2**), Col1A1 (**A3**), FN (**A4**), or SMA (**A5**). Data are the mean values ± SD from 3 independent cultures per treatment. *p<0.05; **p<0.01; ***p<0.001; ****p<0.001, one-way ANOVA with Tukey correction for multiple comparisons. Red boxes highlight the rescue effect of siGαs on CTX-mediated inhibition of TGF-β-induced expression of Col1A1, FN, and SMA.

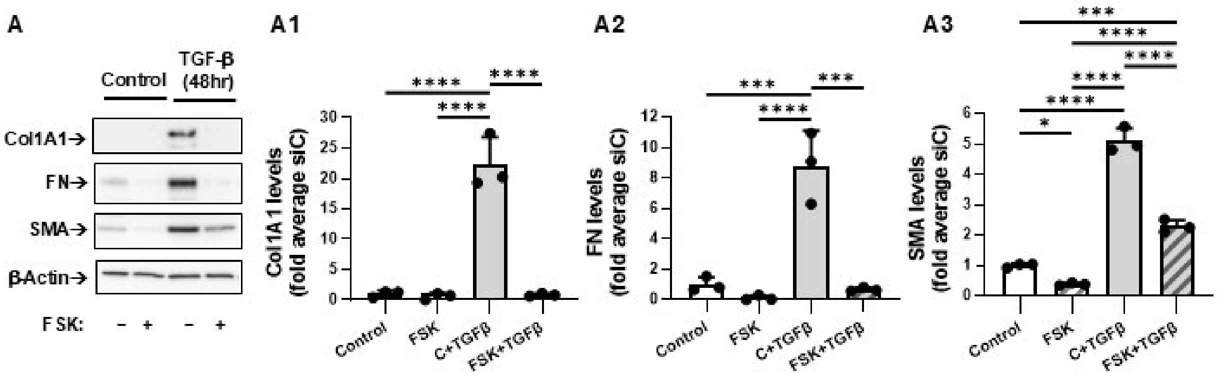

The adenylyl cyclase activator forskolin attenuates TGF-β-induced myofibroblast differentiation.
HLF were serum starved for 48 hours and then treated with either vehicle or 10 μM forskolin (FSK), immediately followed by treatment with either vehicle or 1 ng/ml TGF-β for 48 h as indicated. HFL lysates were analyzed by western blotting using antibodies recognizing collagen 1A1 (Col1A1), fibronectin (FN), smooth muscle cell α-actin (SMA), and β-actin. Shown are representative western blot images (**A**) and quantifications of immunoluminescence signal for Col1A1 (**A1**), FN (**A2**), and SMA (**A3**). Data are the mean values ± SD from 3 independent cultures per treatment. *p<0.05; ***p<0.001; ****p<0.001, one-way ANOVA with Tukey correction for multiple comparisons.

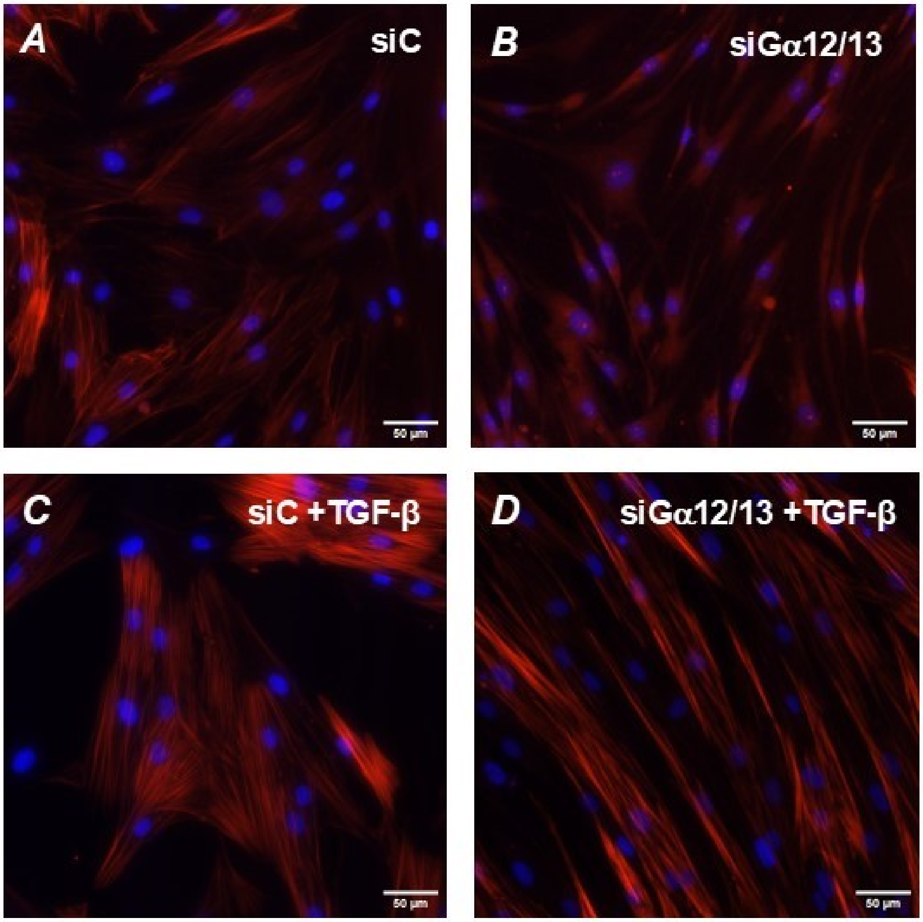

Knockdown of Gα12 and Gα13 partially reduces TGF-β-induced formation of SMA-positive stress fibers in HFL. Representative immunofluorescence SMA images of HLF transfected overnight with control siRNA (siC, 10 nM) or siRNA targeting Gα12 plus Gα13 (5 nM each). HFL were transfected with siRNA overnight, serum-starved for additional 48 h, and the additionally treated for 48 h with either vehicle (***A***, ***C***) or TGF-b (***B***, ***D***). Images were taken using Nikon Ti-2 fluorescent microscope with 20× lens magnification.

Supplemental Figure S5 ● E.B. Reed et al

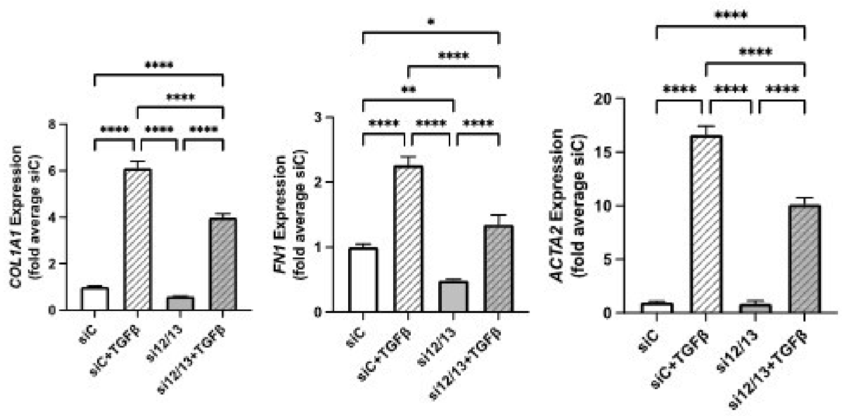

Supplemental Figure S5. Combined knockdown of Gα12 and Gα13 attenuates TGF-β-induced mRNA levels of *Col1A1, FN and SMA.* HLF were transfected siC (10 nM) or with a combination of siGα12 (5nM) + siGα13 (5nM) overnight, serum starved for 48 hours, and treated with vehicle or 1 ng/ml TGF-β for 24 hours. mRNA levels for Col1A1, FN1, ACTA2 (SMA) were measured by real time 1PCR and normalized to the levels of housekeeping ribosomal RPL13 mRNA as indicated. Data are the mean values ± SD from 3 independent cultures per treatment. *p<0.05, **p<0.01; ***p<0.001; ****p<0.001, one-way ANOVA with Tukey correction for multiple comparisons.

Supplemental Figure S6 ● E.B. Reed et al. (new, ungrouped panels)

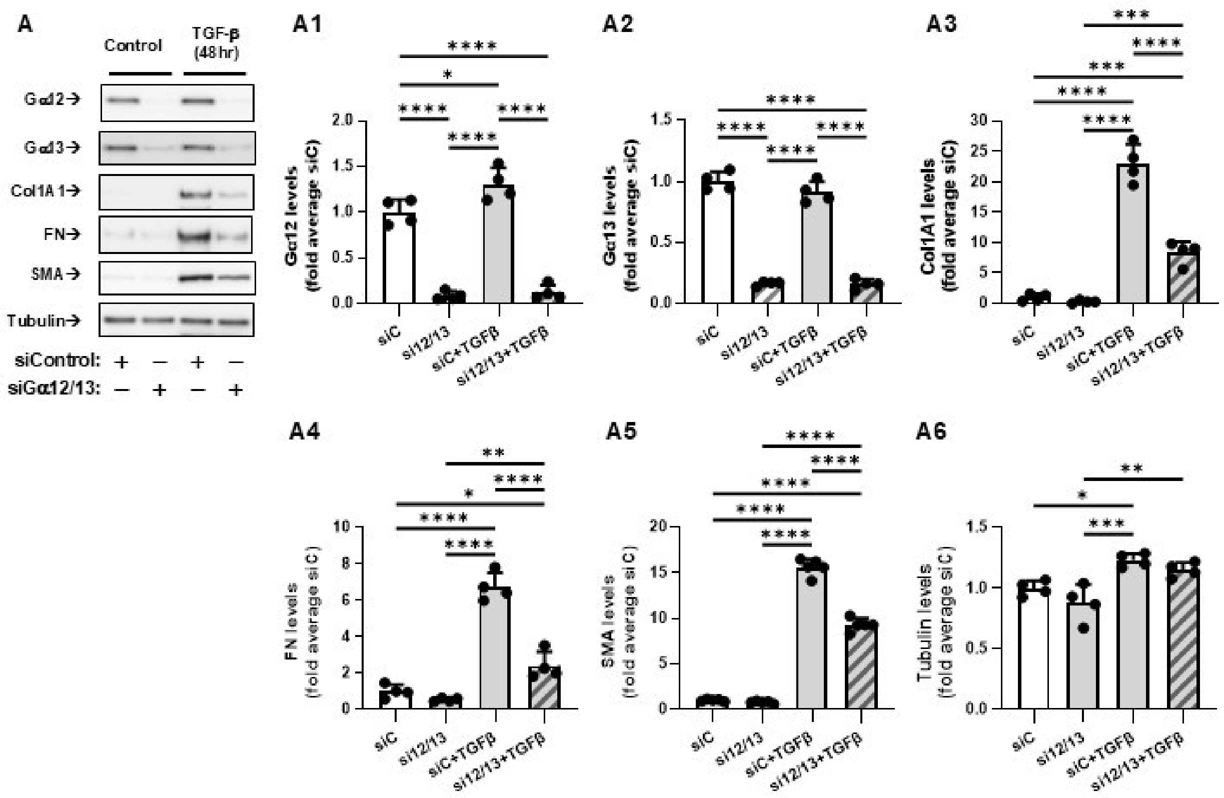

Inhibition of the TGF-β-induced myofibroblast differentiation by a combined knockdown of Gα12 and Gα13 using alternative siRNA constructs.

A, Representative images and quantification of western blot analyses of HLF transfected with control siRNA (siC), or a combination of siRNAs targeting siGα13 and siGα13. HLF were transfected overnight with either siC (10nM), or a combination of siGα12 (GNA12_3, 5nM) and Gα13 (GNA13_6, 5nM). HFL were then serum starved for 48 h and additionally treated for next 48 h with either vehicle or 1 ng/ml TGF-β. Protein lysates were analyzed by western blotting using antibodies recognizing Gα12 (**A1**), Gα13 (**A2**), Col1A1 (**A3**), FN (**A4**), SMA (**A5**), or tubulin (**A6**). Data are the mean values ± SD from 4 independent cultures per treatment. *p<0.05, **p<0.01; ***p<0.001; ****p<0.001, one-way ANOVA with Tukey correction for multiple comparisons.

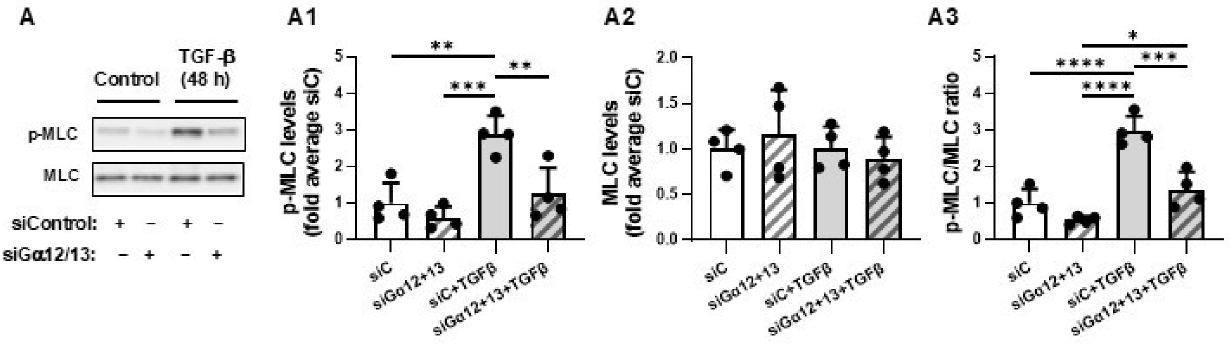

Combined knockdown of Gα12 and Gα13 inhibits TGF-β-induced phosphorylation of myosin light chain (MLC). Representative images and quantification of western blot analyses of HLF transfected overnight with either control siRNA (siC, 10nM) or a combination of siRNAs targeting Gα12 (5nM) and Gα13 (5nM). HLF were then serum starved for 48 h and additionally treated with either vehicle or 1 ng/ml TGF-β for other 48 h. Protein lysates were analyzed using antibodies recognizing p-MLC or total MLC. Quantifications show luminescence levels of p-MLC (**A1**), total MLC (**A2**), or ratios of p-MLC/MLC (**A3**). The relative luminescence values were normalized to the average of siC samples. Data are the mean values ± SD from 4 independent cultures per treatment. *p<0.05; **p<0.01; ***p<0.001; ****p<0.001, one-way ANOVA with Tukey correction for multiple comparisons.

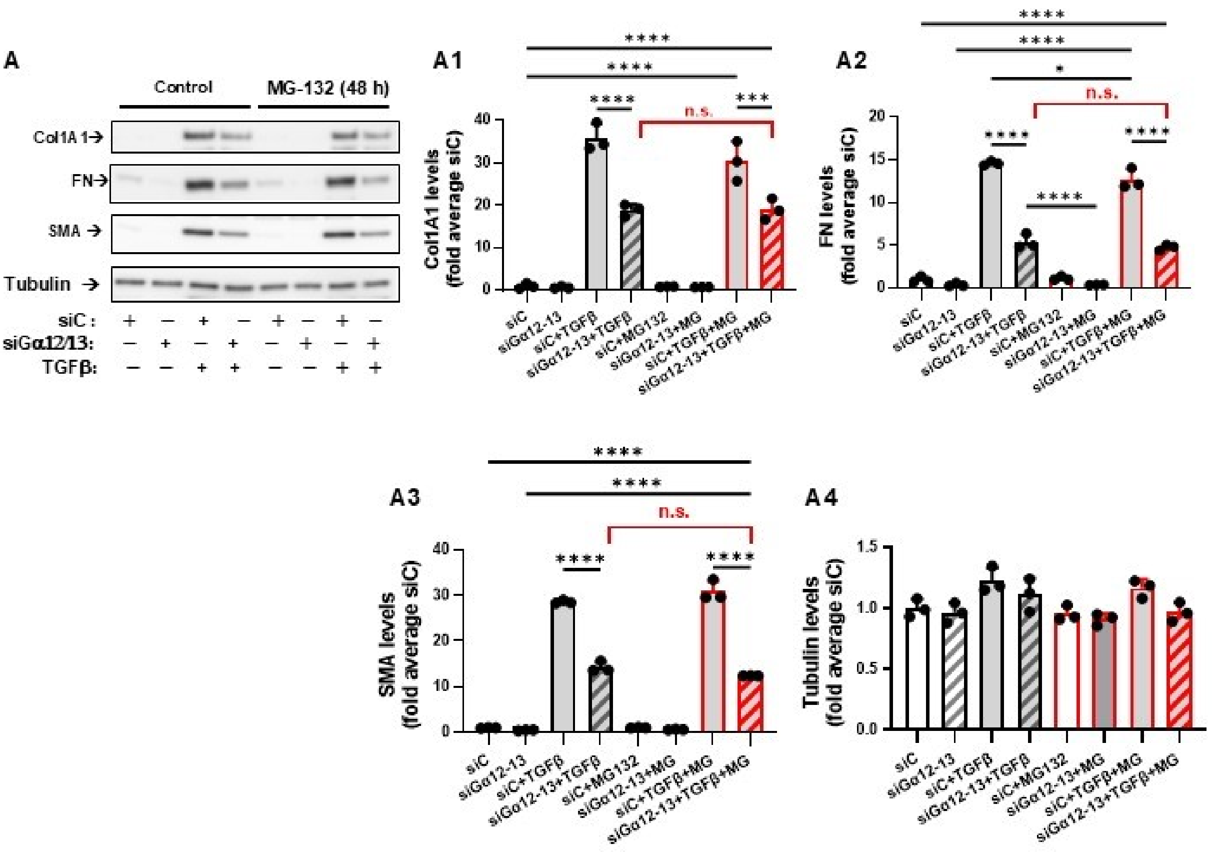

Proteasome inhibitor MG-132 has marginal effect on the TGF-β-induced myofibroblast differentiation and does not rescue from the inhibitory effect of Gα12/13 knockdown.

HLF were transfected overnight with either control siRNA (siC, 10 nM) or the combination of siRNAs targeting Gα12 (5nM) and Gα13 (5nM). HLF were then serum starved for 48 hours and next treated with vehicle, 3 μM MG-132, and/or 1 ng/ml TGF-β for additional 48 h. Protein lysates were analyzed with antibodies against Col1A1, FN, SMA and tubulin as indicated. Shown are representative western blot images and quantifications of chemiluminescent signal for Col1A1, FN, SMA, and tubulin as indicated. Data are the mean values ± SD from 3 independent cultures per treatment. *$p < 0.05$; ****$p < 0.001$, one-way ANOVA with Tukey correction for multiple comparisons. For the purpose of clarity, only physiologically important statistical values are presented.

Supplemental Figure S9 ● E.B. Reed et al.

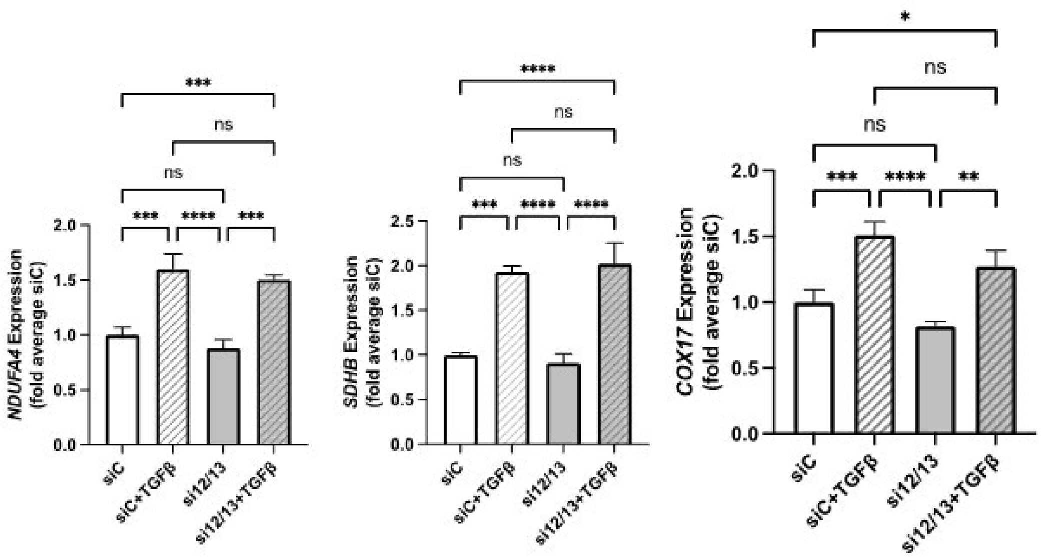

Combined knockdown of Gα12 and Gα13 does not affect the basal or TGF-β-induced mRNA levels of mitochondrial metabolic genes. HLFs were transfected with control siRNA (siC, 10 nM) or with a combination of siRNAs targeting Gα12 (5nM) and Gα13 (5nM) overnight. Cells were then serum starved for 48 hours and additionally treated with vehicle or 1 ng/ml TGFβ for 24 hours. mRNA levels of NDUFA4, SDHB and COX17 were measured by real time qPCR and normalized to the levels of RPL13 housekeeping gene as indicated. Data are the mean values ± SD from 3 independent cultures per treatment. *p<0.05, **p<0.01; ***p<0.001; ****p<0.001, one-way ANOVA with Tukey correction for multiple comparisons.

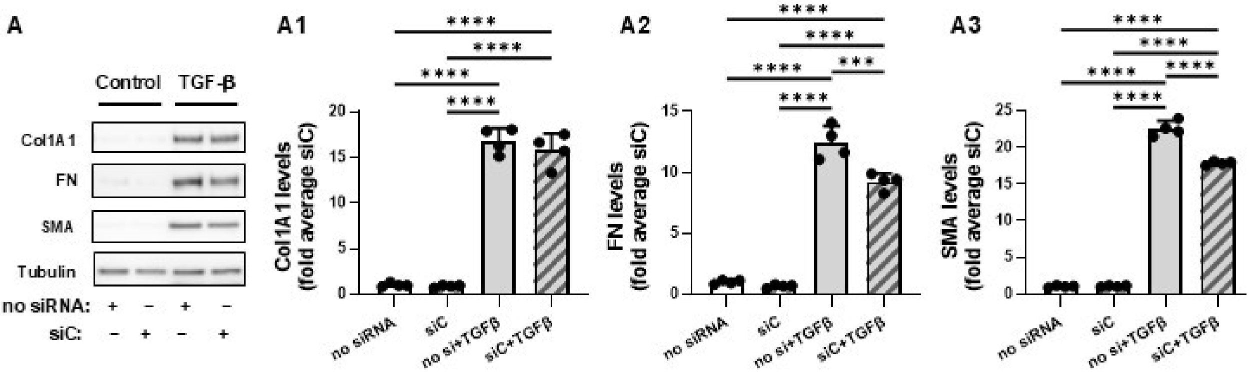

Transfection with negative control siRNA has marginal effect on the TGF-β-induced myofibroblast differentiation. HLF were exposed for 24 h to either transfection reagent alone (no siRNA) or control siRNA (siC, 10 nM). HLF were then serum starved for 48 hours, following by treatment with either vehicle or 1 ng/ml TGF-β for additional 48 hours. **A,** Cell lysates were analyzed by western blotting using antibodies against Col1A1, FN, SMA, and tubulin as indicated. Quantifications of immunoreactivity levels of Col1A1 **(A1)**, FN (**A2)** or SMA (**A3**) are also shown: the relative expression values were normalized to the average values in "no siRNA" samples. Data are the mean values ± SD from 4 independent cultures per treatment. ***$p<0.001$; ****$p<0.001$, one-way ANOVA with Tukey correction for multiple comparisons.

Supplemental Figure S11 ● E.B. Reed et al.

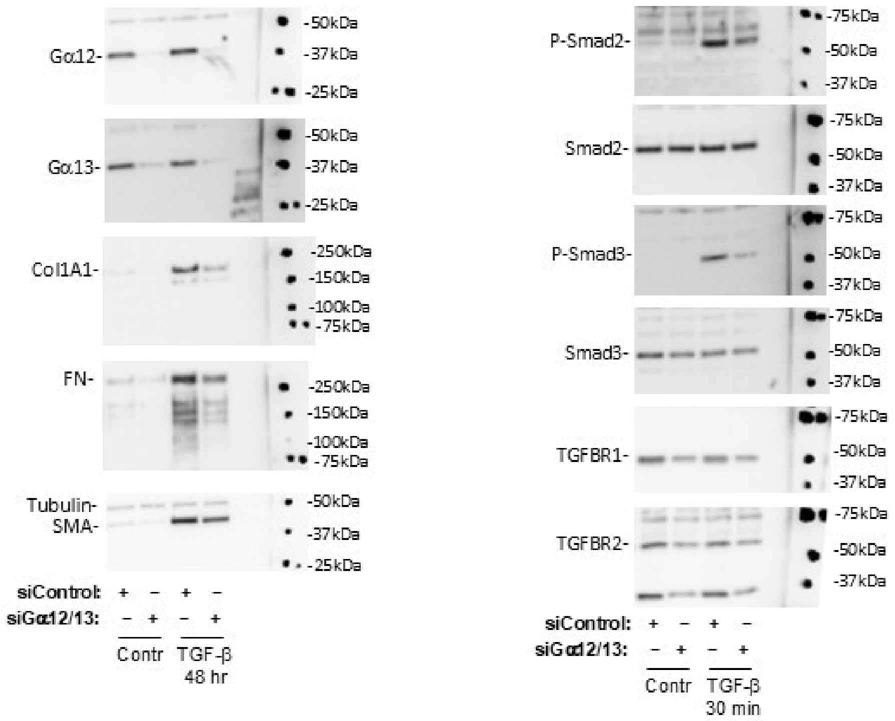

Figure S11. Original western blot images for relevant proteins, with molecular weight markers.

Supplemental Figure S12. Sequences for RT-qPCR analyses of mRNA expression:

hFN1F504 GGA GGA AGC CGA GGT TTT AAC TG

hFN1R607 CAG CTT ATT CTC CCT CGC CC

hACTA2F1040 GCA CCA TGA AGA TCA AGA TCA TTG CCC

hACTA2R1230 GCT AGA GAC AGA GAG GAG CAG G

hCol1A1F4345 CGT CAC TGT CGA TGG CTG CAC

hCol1A1R4535 GCC AGG TTG GGA TGG AGG G

NDUFA4- F: 5'-TCTCTTGCGTCTGGCATTGT-3', R: 5'-TGGGACCCAGTTTGTTCCAG-3'

SDHB- F: 5'- CACTCTAGCTTGCACCCGAA-3', R: 5'-ACATGTGTGGAAGAGGGTAGA-3'

COX17- F: 5'-GTGGTCGGGTCTCTGTTGAC-3', R: 5'-AAGCTTGCCGTTCTCCTCTC-3'

RPL13- F: 5'-GTCGTACGCTGTGAAGGCAT-3', R: 5'-GGAAAGCCAGGTACTTCAACTT-3
